# Changes in Intestinal Flora Structure and Metabolites Are Associated With Myocardial Fibrosis in Patients With Persistent Atrial Fibrillation

**DOI:** 10.3389/fnut.2021.702085

**Published:** 2021-08-23

**Authors:** Langsha Liu, Juan Su, Rui Li, Fanyan Luo

**Affiliations:** ^1^Department of Cardiac Surgery, Xiangya Hospital, Central South University, Changsha, China; ^2^Department of Medical Administration, Zhuzhou Central Hospital, Zhuzhou, China; ^3^Operating Theatre, Zhuzhou Central Hospital, Zhuzhou, China

**Keywords:** persistent atrial fibrillation, intestinal flora, metabolism, myocardial fibrosis, gut-heart

## Abstract

**Background:** The occurrence of atrial fibrillation is often accompanied by myocardial fibrosis. An increasing number of studies have shown that intestinal flora is involved in the occurrence and development of a variety of cardiovascular diseases. This study explores the relationship between changes in the structure and function of intestinal flora and the progression of myocardial fibrosis in patients with persistent atrial fibrillation.

**Methods:** Serum and stool samples were collected from 10 healthy people and 10 patients with persistent atrial fibrillation (PeAF), and statistical analyses were performed on the subjects' clinical baseline conditions. ELISA was used to measure the levels of carboxy-terminal telopeptide of type I collagen (CTX-I), propeptide of type I procollagen (PICP), procollagen III N-terminal propeptide (PIIINP), fibroblast growth factor-23 (FGF-23), and transforming growth factor-beta 1 (TGF-β1) in serum. Through 16S rRNA sequencing technology, the structural composition of the intestinal flora was detected and analyzed. In addition, metabolomics data were analyzed to determine the differences in the metabolites produced by the intestinal flora of the subjects.

**Results:** By comparing the baseline data of the subjects, it was found that compared with those of the control group, the levels of creatinine (CRE) and serum uric acid (SUA) in the serum of PeAF patients were significantly increased. In addition, we found that the levels of CTX-I, PICP, PIIINP, and TGF-β1 in the serum of PeAF patients were significantly higher than those of the control group subjects. Although the control and PeAF groups exhibited no significant differences in the α diversity index, there were significant differences in the β diversity indexes (Bray-Curtis, weighted UniFrac and Anosim). At the phylum, family and species levels, the community structure and composition of the intestinal flora of the control group and those of the PeAF group showed significant differences. In addition, the compositions of the intestinal metabolites in the two different groups of people were significantly different. They were correlated considerably with PIIINP and specific communities in the intestinal flora.

**Conclusion:** Pathologically, PeAF patients may have a higher risk of myocardial fibrosis. Systematically, abnormal changes in the structure and composition of the intestinal flora in PeAF patients may lead to differences in intestinal metabolites, which are involved in the process of myocardial fibrosis through metabolite pathways.

## Introduction

Atrial fibrillation (AF) is one of the most common arrhythmias, and persistent atrial fibrillation (PeAF) is often associated with a higher risk of stroke (1). Atrial fibrosis is considered a potential key factor and biomarker in the treatment of atrial fibrillation (2). Atrial fibrosis plays an essential role in the occurrence and continuation of atrial fibrillation through structural and electrical remodeling (3). In animal models, this heterogeneity can affect electrical conduction and signal transmission between cells, thereby providing a basis for the occurrence and development of atrial fibrillation (4). Myocardial fibrosis in atrial fibrillation is mediated by various factors, but the specific mechanism of atrial fibrosis-atrial fibrillation is still not fully understood.

Imbalance in collagen synthesis and its decomposition and metabolism are the causes of the occurrence and development of myocardial fibrosis (5). It is well-known that the extracellular matrix of the heart is mainly composed of fibrous type I collagen and type III collagen. Many circulating biomarkers related to collagen synthesis have been proposed to assess myocardial fibrosis, such as CTX-I, PICP, and PIIINP. They are considered functional factors that directly reflect the degree of fibrosis (6). In addition, FGF-23 directly participates in the development of myocardial fibrosis by activating fibroblast growth factor receptor (FGFR) (7). Studies have also shown that FGF-23 induces atrial fibrosis in patients with atrial fibrillation by increasing reactive oxygen species (ROS) production and subsequently activating signal transducer and activator of transcription 3 (STAT3) and SMAD3 signaling (8). Therefore, FGF-23 can also be used as a marker of myocardial fibrosis. Besides, TGF-β1, as an essential fibrosis mediator, promotes the synthesis of collagen fibers through typical Smad-dependent and non-classical Smad-independent pathways and can also be used as an indirect marker of myocardial fibrosis (9, 10).

As the concept of the “gut-heart” axis has gradually attracted attention, increasing evidence has confirmed the connection between the gut microbiota and cardiovascular diseases (CVDs), such as hypertension, atherosclerosis, myocardial infarction, heart failure, and arrhythmia (11, 12). Atrial fibrillation has also been determined to be related to an imbalanced intestinal flora (13). For example, Zuo et al. found that the duration of persistent atrial fibrillation is related to changes in human intestinal flora and metabolic phenotypes (14). In addition, studies have found that specific intestinal microbial changes (such as changes in the abundances of Nitrosomonadaceae and Lentisphaeraceae) are associated with the risk of atrial fibrillation recurrence (15). Therefore, we reasonably speculate that the intestinal flora may be involved in atrial fibrillation myocardial fibrosis development. Intestinal microbial-derived metabolites, such as trimethylamine N-oxide (TMAO), short-chain fatty acids (SCFAs), and secondary bile acids (BAs), have been proposed to be markers of major cardiac adverse events (16). A recent study showed that TMAO synthesized by the gut microbiota is enriched in patients with atrial fibrillation (17). The underlying mechanism of the intestinal flora is generally thought to involve immune regulation, host energy metabolism, and oxidative stress. These findings indicate that the function of the gut microbiome is similar to that of an endocrine organ, which can directly or indirectly affect the physiology of the host by producing biologically active metabolites (18). Therefore, we speculate that the abnormal changes in the intestinal flora in PeAF patients may lead to disordered or imbalanced host-related metabolic function, which may be one of the crucial mechanisms of the intestinal flora in the mediation of myocardial fibrosis.

In this study, based on 16S rRNA sequencing and metabonomic techniques, we investigated the correlation between atrial fibrosis and gut microbiota and their derived metabolites in PeAF patients. The mechanism of atrial fibrosis in PeAF patients was preliminarily explored from the perspective of “gut-heart.” This study may provide new ideas for the prevention and treatment of persistent atrial fibrillation.

## Materials and Methods

### Subjects

Ten PeAF patients admitted to the Cardiac Surgery Department of Xiangya Hospital were selected as the PeAF group. Ten volunteers with a healthy physical examination in the same period were selected as the control group. There were no statistically significant differences in sex or age between the two groups of people (*P* > 0.05). The selection criteria for PeAF patients were based on the guidelines for atrial fibrillation recommended by the European Society of Cardiology (ESC) (19). The exclusion criteria were as follows: (1) patients with gastrointestinal diseases or recent diarrhea and (2) patients who had recently taken antibiotics, hormone drugs, or microecological preparations. Clinical baseline condition for all subjects was obtained by checking hospital or medical records. After standing for 2 h, all the collected blood from subjects was centrifuged at 4,000 rpm for 15 min to obtain serum. Architect CI8200 integrated system (Abbott, IL, USA) was used to determine concentrations of CRE and SUA in all collected volunteer serum (20). This study was approved by the Ethic Committee of Xiangya Hospital Central South University (202004176), and the patients and family members consented to their inclusion in the study.

### Sample Selection

All participants used a sterile stool collection kit to collect stool samples. After the sample was collected, it was quickly placed in a freezing tube, placed in liquid nitrogen for quick freezing, and then transferred to a −20°C refrigerator for storage. Five ml of fasting venous blood was collected from each subject, maintained at room temperature for 2 h, and then centrifuged at 2–8°C at 1,000 g for 15 min. The supernatant was then collected for subsequent experiments.

### ELISA

According to the manufacturer's instructions, ELISA was performed using the following kits: CTX-I (CSB-E11224h, CUSABIO BIOTECH, Wuhan), PICP (CSB-E08079h, CUSABIO BIOTECH, Wuhan), PIIINP (JL19037, Jianglai, China), FGF-23 (CSB-E10113h, CUSABIO BIOTECH, Wuhan), and TGF-β1 (CSB-E04725h, CUSABIO BIOTECH, Wuhan). After the reactions were terminated, the optical density (OD value) of each sample was sequentially measured at 450 nm wavelength with a microplate reader.

### 16S rRNA Sequencing

Illumina NovaSeq PE250 was used for 16S amplicon sequencing to obtain raw data. The raw data were subjected to joint removal, filtering, deduplication, base correction, and removal of chimera sequences to obtain clean data for subsequent analyses. The QIIME 2 analysis process was adopted, and DADA2 was used to denoise the raw data, equivalent to clustering with 100% similarity. Only low-quality sequences were removed and corrected, and algorithms were identified and de-embedded. The denoised sequences were de-redundant. Additionally, feature [including Amplifier sequencing variation (ASV)] information was obtained. Each ASV sequence was annotated to obtain corresponding species information, including abundance distribution. QIIME 2 software was used to calculate the alpha diversity index (Chao1, ACE, Shannon, Simpson) of each sample. The ANOSIM analysis method was used to test the significance of differences in the community structures of the grouped samples. R software was used to draw a PCoA dimensionality reduction analysis diagram based on Bray-Curtis, unweighted UniFrac, and weighted UniFrac distances (phyloseq/vegan package).

### Metabolomics

After freezing and grinding each stool sample, ~50 mg was weighed and placed in a centrifuge tube. Four hundred microliter precooled (4°C) extraction solution (Methanol: Acetonitrile (v/v) = 1:1) was added to the sample tube, followed by vortexing and mixing, and then the mixed solution was incubated on ice for 10–15 min. Subsequently, the mixture was centrifuged at 16,000 g at 4°C for 10 min, and the supernatant was collected, transferred to a new centrifuge tube, and dried with nitrogen. The LC-MS analysis system consisted of an ultrahigh-performance liquid chromatograph (Agilent 7890B-5977B) paired with a Q Exactive Orbitrap high-resolution mass spectrometer (Thermo Fisher Scientific). The flow rate was set at 0.3 ml/min; the temperature of the sample tray was 4°C, and the column temperature was 40°C. A Waters HSS T3 column (100 × 2.1 mm, 1.7 μm) was used with (A) H_2_O (0.1% formic acid) and (B) acetonitrile, with an injection volume of 3 μl. Mass conditions were as follow: Time of Flight is 60–100 dm/z, Ion source Gas1is 55psi, Ion source Gas2 is 55psi, Curtain Gas is 35psi, Temperature is set to 550°C, Declustering potential is 80 V, Collision Energy is 10 V, IonSpray Voltage is 5500 V (POS) or −4500 V (NEG). According to the plain peak area obtained by detection from the detection and AB SCIEX (AB Sciex Pte Ltd.) commercial and self-built databases, relative quantification of metabolites was carried out. The signal correction of the LC-MS metabolomics raw data was performed by using the R language package Stattarget. Then, we use MetaboAnalyst 5.0 (https://www.metaboanalyst.ca/faces/home.xhtml) website online analysis for a series of subsequent analyses, including Principal Component Analysis (PCA), partial least-squares discrimination analysis (PLS-DA), orthogonal partial least-squares discrimination analysis (OPLS-DA), and Sparse PLS discriminant analysis (sPLS-DA). Kyoto Encyclopedia of Genes and Genomes (KEGG, https://www.genome.jp/kegg/pathway.html) database was used for metabolic pathway analysis.

### Data Analyses

GraphPad (GraphPad Software, San Diego, California, USA) statistical software was used for the analyses. Variables conforming to a normal distribution are expressed as the mean ± standard deviation (SD). Comparisons between two groups were performed using *t*-tests or one-way analysis of variance (ANOVA). The measurement data that did not conform to a normal distribution were analyzed using the Wilcoxon rank-sum test. The Spearman correlation analysis method was used for correlation analysis. A *p* < 0.05 was considered significantly different.

## Results

### Baseline Characteristics of the Subjects

First, we analyzed and compared the baseline characteristics of the 10 healthy people and the 10 PeAF patients included in this study. As shown in Table 1, there were no significant differences between the two groups in terms of age, body mass index (BMI), systolic blood pressure (SBP), diastolic blood pressure (DBP), fasting blood glucose (FBG), albumin (ALB), total cholesterol (TC), triacylglycerol (TG), high-density lipoprotein cholesterol (HDL-c), low-density lipoprotein cholesterol (LDL-c), white blood cells (WBC) or neutrophil (N). However, we noticed that the levels of CRE and SUA in the serum of the PeAF patients were significantly higher than that of the healthy individuals.

**Table 1:** Baseline characteristics of subjects.

	**Control**	**PeAF**	**Difference**	***P*-value**
Age	40.5 ± 8.7^a^	71.4 ± 10.2	ns	0.673
Female/Male	6/4	6/4		
BMI/(kg/m^2^)	22.24 ± 2.30	22.118 ± 3.00	ns	>0.9999
SBP/mmHg	124 ± 11.08	128 ± 24.47	ns	>0.9999
DBP/mmHg	72 ± 7.28	84.2 ± 13.57	ns	>0.9999
**NYHA**				
II		3		
III		4		
IV		3		
CRE/(μmol/L)	68.9 ± 9.22	144.2 ± 153.20	^***^	0.0003
SUA/(μmol/L)	293 ± 70.85	413.51 ± 111.97	^****^	<0.0001
FBG/(μmol/L)	5.039 ± 0.49	6.769 ± 2.30	ns	>0.9999
ALB/(g/L)	48.41 ± 3.64	35.35 ± 3.84	ns	0.9998
TC/(μmol/L)	4.942 ± 0.96	3.77 ± 0.86	ns	>0.9999
TG/(μmol/L)	1.048 ± 0.41	1.149 ± 0.43	ns	>0.9999
HDL-c/(μmol/L)	1.329 ± 0.32	1.01 ± 0.48	ns	>0.9999
LDL-c/(μmol/L)	3.164 ± 1.01	2.182 ± 0.63	ns	>0.9999
WBC/(×10^9^/L)	5.776 ± 1.10	6.151 ± 2.87	ns	>0.9999
N/(×10^9^/L)	3.063 ± 0.51	4.795 ± 2.73	ns	>0.9999

a *represented mean ± standard deviation, ns indicated no statistical difference*,

*** *compared with the control group, P < 0.001*,

**** *compared with the control group, P < 0.0001. BMI, Body Mass Index; SBP, Systolic Blood Pressure; DBP, Diastolic Blood Pressure; NYHA, New York Heart Association class; CRE, Creatinine; SUA, Serumuric Acid; FBG, Fasting Blood Glucose; ALB, Albumin; TC, Total cholesterol; TG, Triacylglycerol; HDL-c, high-density lipoprotein cholesterol; LDL-c, low-density lipoprotein cholesterol; WBC, White Blood Cells; N, Neutrophil*.

### Expression of Cardiac Fibrosis Markers

**Figure 1:**
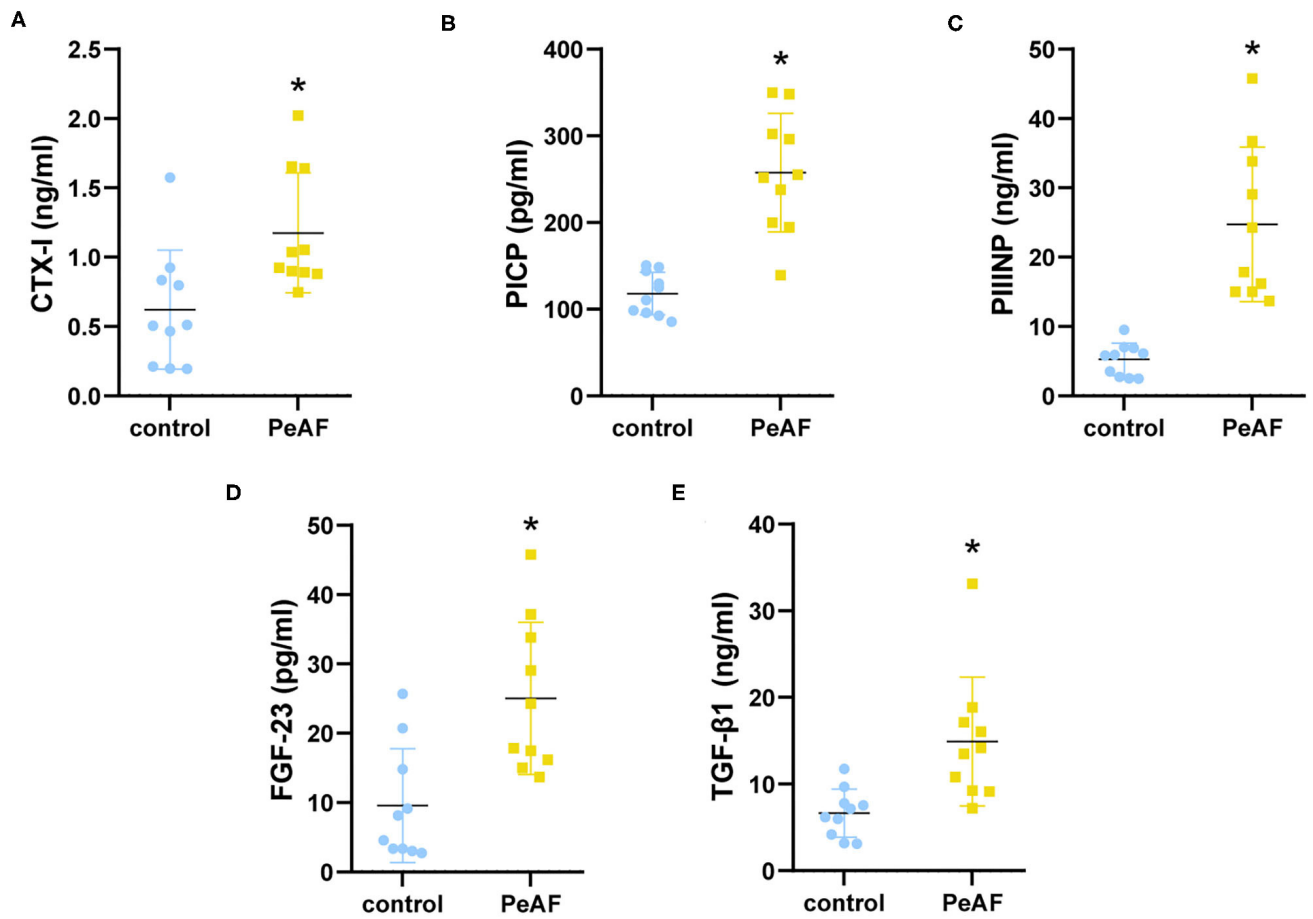
Serum levels of myocardial fibrosis markers. **(A)** Levels of CTX-I in serum. **(B)** Levels of PICP in serum. **(C)** Levels of PIIINP in serum. **(D)** Levels of FGF-23 in serum. **(E)** Levels of TGF-β1 in serum. **(F)** Levels of PIIINP in serum. *p < 0.05 vs. control group. CTX-I, carboxy-terminal telopeptide of type I collagen; PICP, propeptide of type I procollagen; PIIINP, procollagen III N-terminal propeptide; FGF-23, fibroblast growth factor-23; TGF-β1, transforming growth factor-beta 1.

We first detected the expression of cardiac fibrosis markers in the serum of all the subjects. As shown in Figure 1A, compared with healthy people, the level of CTX-I was increased in the serum of the PeAF patients. Similarly, the levels of PICP and PIIINP were also high in the serum of the PeAF patients (Figures 1B,C). In addition, we observed that the level of FGF-23 in the serum of the PeAF patients was significantly higher than that of the healthy people (Figure 1D). Similarly, the content of TGF-β1 in serum of PeAF patients was much higher than that of healthy people, as shown in Figure 1E.

### Alterations in Intestinal Flora Diversity

**Figure 2:**
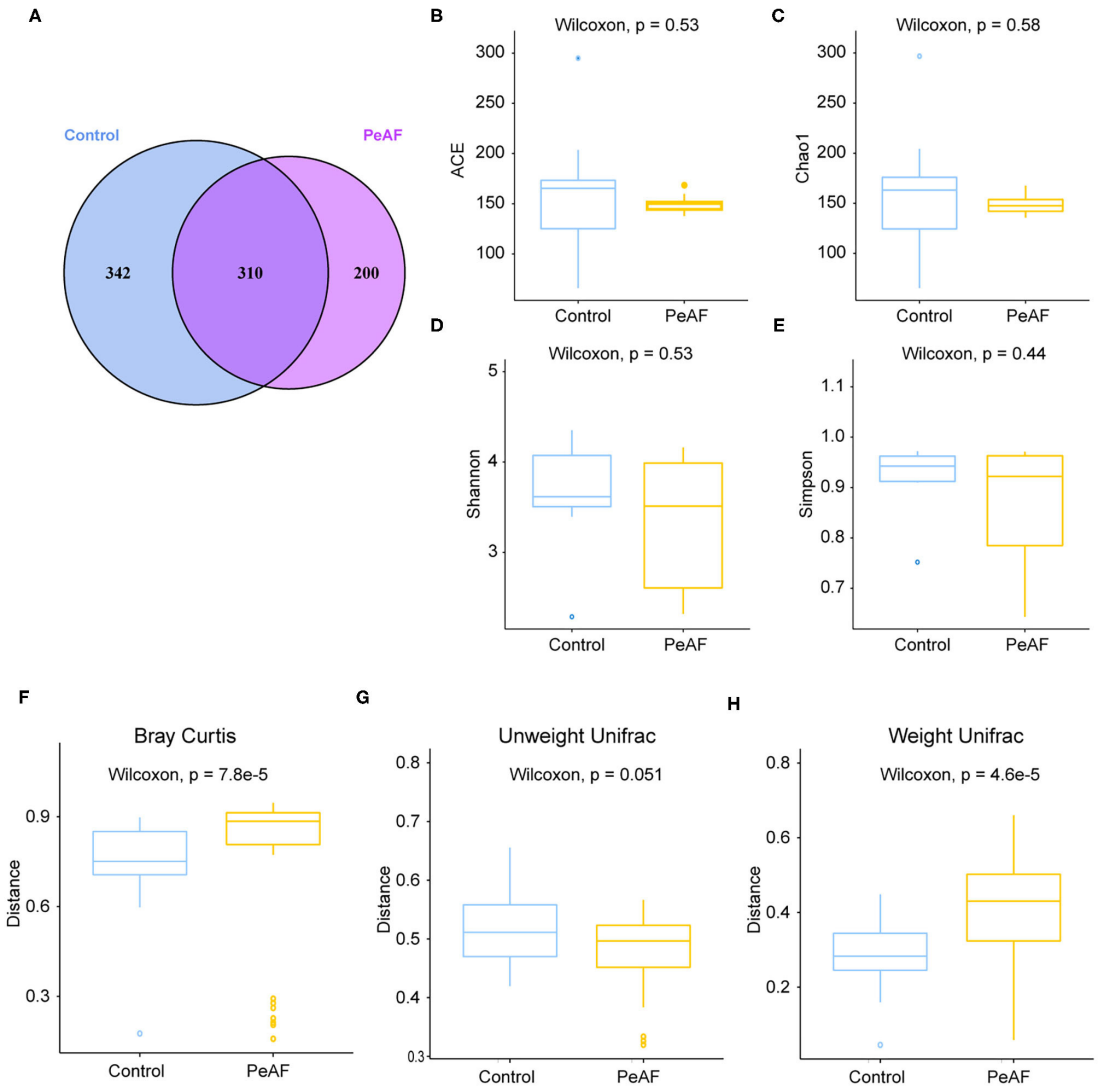
Alterations in intestinal flora diversity. **(A)** Venn diagram of shared ASVs and ASVs unique to different populations. **(B)** ACE index. (**C)** Chao1 index. **(D)** Shannon index. **(E)** Simpson index. **(F)** Bray-Curtis analysis. **(G)** Unweighted UniFrac analysis. **(H)** Weighted UniFrac analysis. Wilcoxon rank sum test; *p* < 0.05 was considered statistically significant. When 0.05 < *p* < 0.1, there was a relevant changing trend.

To further explore the differences between the diversity of intestinal flora in the PeAF patients and healthy people, we performed 16S rRNA sequencing on all subjects. As shown in Figure 2A, a Venn diagram was drawn based on the common and unique ASVs of the two groups of people. There were 342 unique ASVs in the control group, 200 unique ASVs in the PeAF group, and 310 ASVs were shared between the control and PeAF groups. The Wilcoxon rank-sum test was performed to analyze the differences in the alpha diversity index between the two groups. The results showed that the Chao1 index, ACE index, Shannon index and Simpson index showed no significant differences between the PeAF group and the control group (*p* > 0.05) (Figures 2B–E). Therefore, there were no significant differences between the intestinal flora of PeAF patients and healthy people in terms of alpha diversity. Next, we analyzed the differences in Bray-Curtis, unweighted UniFrac, and weighted UniFrac between the two groups using the Wilcoxon rank-sum test. The results showed that Bray-Curtis and weighted UniFrac index were significantly different between the two groups (Figures 2F,H). The unweighted UniFrac index was slightly different between the groups (0.05 < *p* < 0.1) (Figure 2G). These results show significant differences in the β diversity of the intestinal flora between the PeAF patients and healthy people.

### Changes in the Intestinal Microbial Community Structure

**Figure 3:**
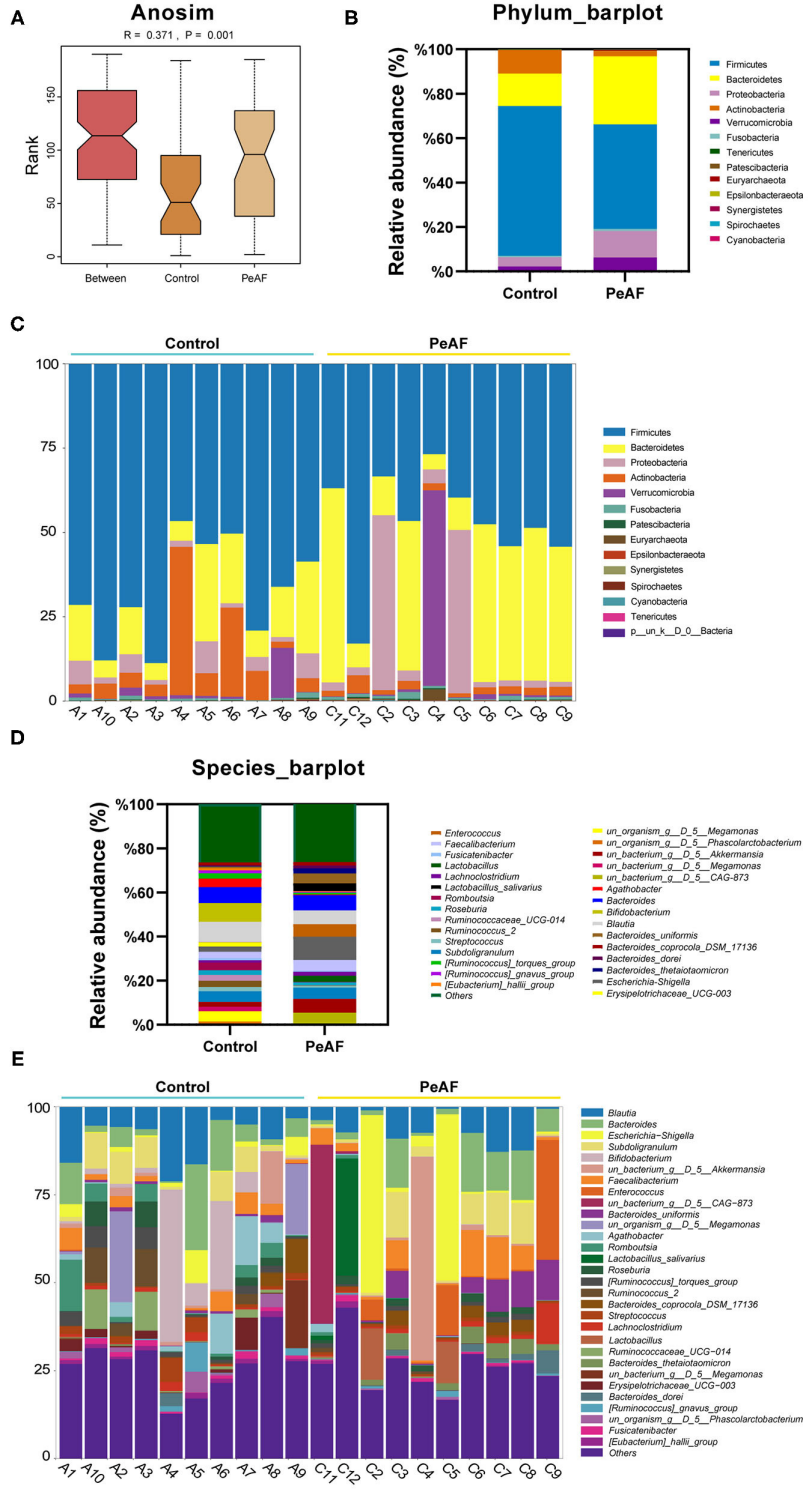
Changes in the intestinal microbial community structure. **(A)** Anosim analysis; *p* < 0.05 was regarded as statistically significant. **(B,C)**. Histogram of the relative abundances in the intestinal flora of different populations at the phylum level. **(D,E)** Species-level analysis of the relative abundances in the intestinal flora of different populations.

As shown in Figure 3A, we used the Anosim analysis method to conduct similarity analysis on the community structures of the grouped samples and found that the community structures between the control group and the PeAF group were significantly different (*P* = 0.001) (Figure 3A). Further analysis of the community structure differences of different samples and groups at the phylum and species levels showed that at the phylum level, the relative abundances of Firmicutes and Actinobacteria showed a downward trend in the PeAF patients compared to the healthy people. At the same time, Bacteroidetes, Verrucomicrobia, and Proteobacteria followed an opposite trend (Figures 3B,C). At the species level, we noticed that the healthy people had more abundant intestinal flora than the PeAF patients in terms of community structure (Figures 3D,E). Therefore, compared with healthy people, the structural composition of the intestinal flora in PeAF patients is changed.

### Abundance Analysis of Differential Bacteria and Their Correlation With Myocardial Fibrosis

**Figure 4:**
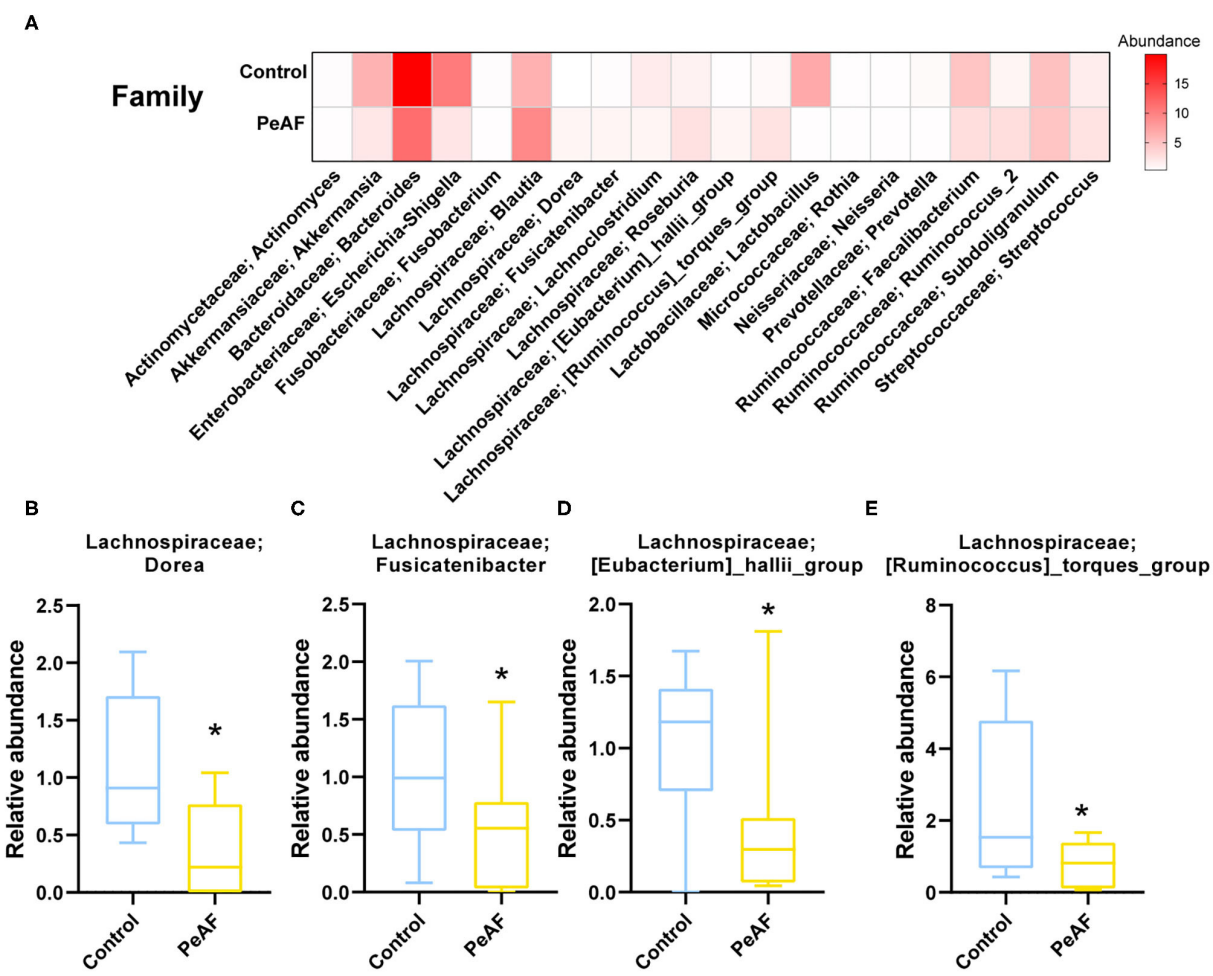
Variations in the abundances in the intestinal flora at the family level. **(A)** Abundance heat map of the top 20 families; the darker the color is, the higher the abundance level. **(B)** The relative abundance of Lachnospiraceae; *Dorea*. **(C)** The relative abundance of Lachnospiraceae; *Fusicatenibacter*. **(D)** The relative abundance of Lachnospiraceae; *[Eubacterium]_hallii_group*. **(E)** The relative abundance of Lachnospiraceae; *[Ruminococcus]_torques_group*. *Compared to the control group, *p* < 0.05.

We further analyzed the differences in the relative abundances in the flora at different levels. As shown in Figure 4A, we first conducted a heat map analysis of the relative abundances of different bacterial groups at the family level. The abundances of different bacterial genera in the two groups of people were quite different. Among these differences, the relative abundances of *Dorea* (Figure 4B), *Fusicatenibacter* (Figure 4C), *[Eubacterium]_hallii_group* (Figure 4D) and *[Ruminococcus]_torques_group* (Figure 4E) were significantly reduced in the PeAF patients. Coincidentally, these four taxa belong to the Firmicutes phylum.

**Figure 5:**
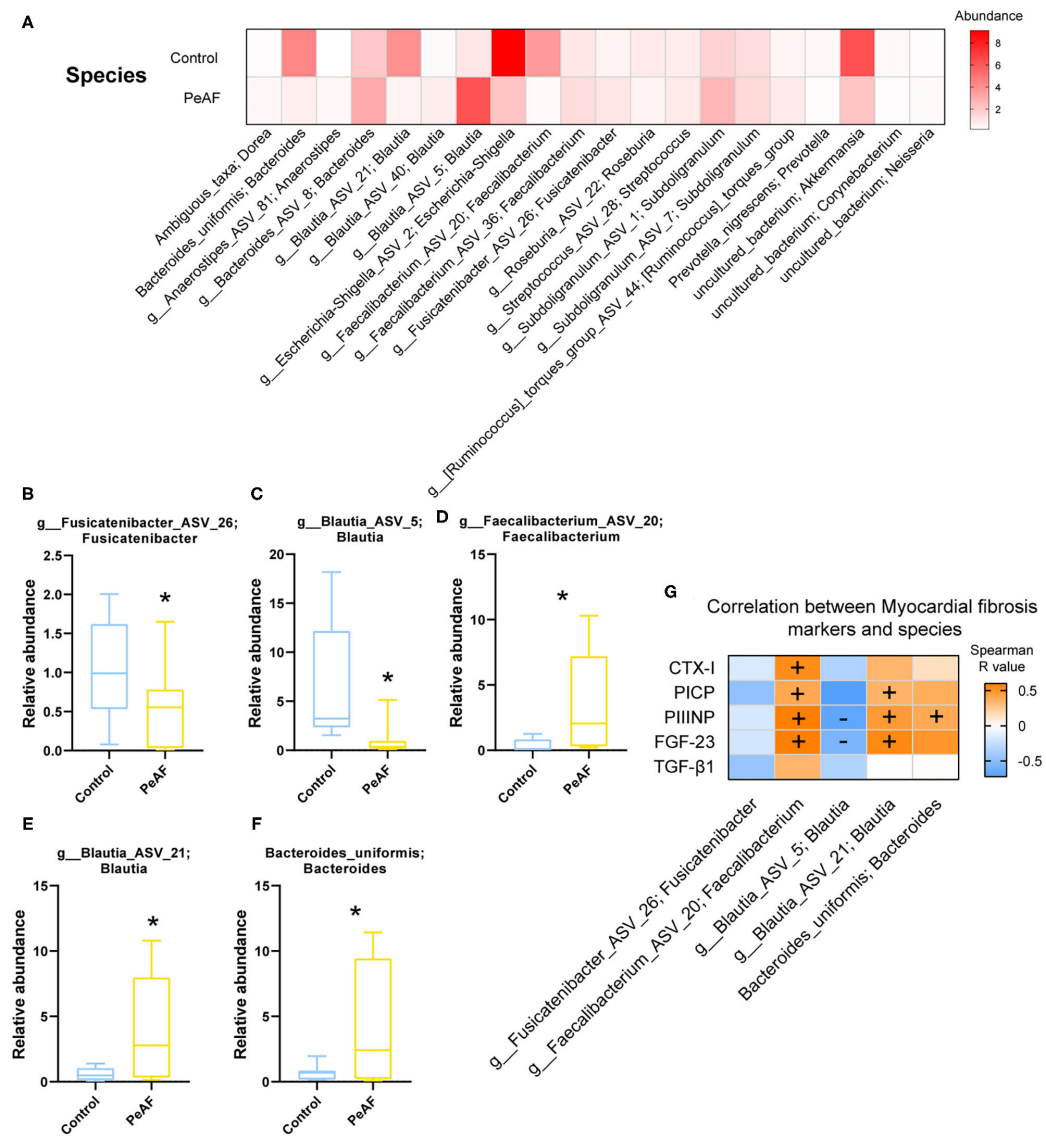
Variations in the abundances in the intestinal flora at the species level. **(A)** Abundance heat map of the top 20 species; the darker the color is, the higher the abundance level. **(B)** Relative abundance of *g_Fusicatenibacter ASV 26; Fusicatenibacter*. **(C)** The relative abundance of *g_Blautia_ASV_5; Blautia*. **(D)** The relative abundance of *g_Faecalibacterium_ASV_20; Faecalibacterium*. **(E)** The relative abundance of *g_Blautia_ASV_21; Blautia*. **(F)** The relative abundance of *Bacteroides uniformis; Bacteroides*. *Compared to the control group, *p* < 0.05. **(G)**. Correlation analysis between myocardial fibrosis markers and different flora. “+” indicates positive correlation (orange), *p* < 0.05. “-” indicates negative correlation (blue), *p* < 0.05.

As shown in Figure 5A, at the species level, the relative abundances of some bacterial taxa were quite different between the control group and the PeAF group. Further analysis revealed that compared with the control group, the abundances of *g_Fusicatenibacter_ASV_26* (*Fusicatenibacter* genus) and *g_Blautia_ASV_5* (*Blautia* genus) were significantly reduced in the PeAF group (Figures 5B,C). The abundances of *g__Faecalibacterium_ASV_20* (*Faecalibacterium* genus), *g__Blautia_ASV_21* (*Blautia* genus), and *Bacteroides_uniformis* (*Bacteroides*) in the PeAF group were significantly increased (Figures 5D–F). In addition, through Spearman's correlation analysis, we found that, at the species level, *g__Faecalibacterium_ASV_20* (*Faecalibacterium* genus) had a significant positive correlation with the CTX-I, PICP, PIIINP and FGF-23 level (*p* < 0.05). *g__Blautia_ASV_21* (*Blautia* genus) was also significantly positively correlated with PICP, PIIINP and FGF-23 levels (*p* < 0.05). *Bacteroides uniformis* (*Bacteroides* genus) was positively correlated with the level of the myocardial fibrosis marker FGF-23 (*p* < 0.05), while *g__Blautia_ASV_5* (*Blautia* genus) was significantly negatively correlated with FGF-23 and PIIINP level (*p* < 0.05) (Figure 5G).

### Difference Analysis of Intestinal Metabolites

**Figure 6:**
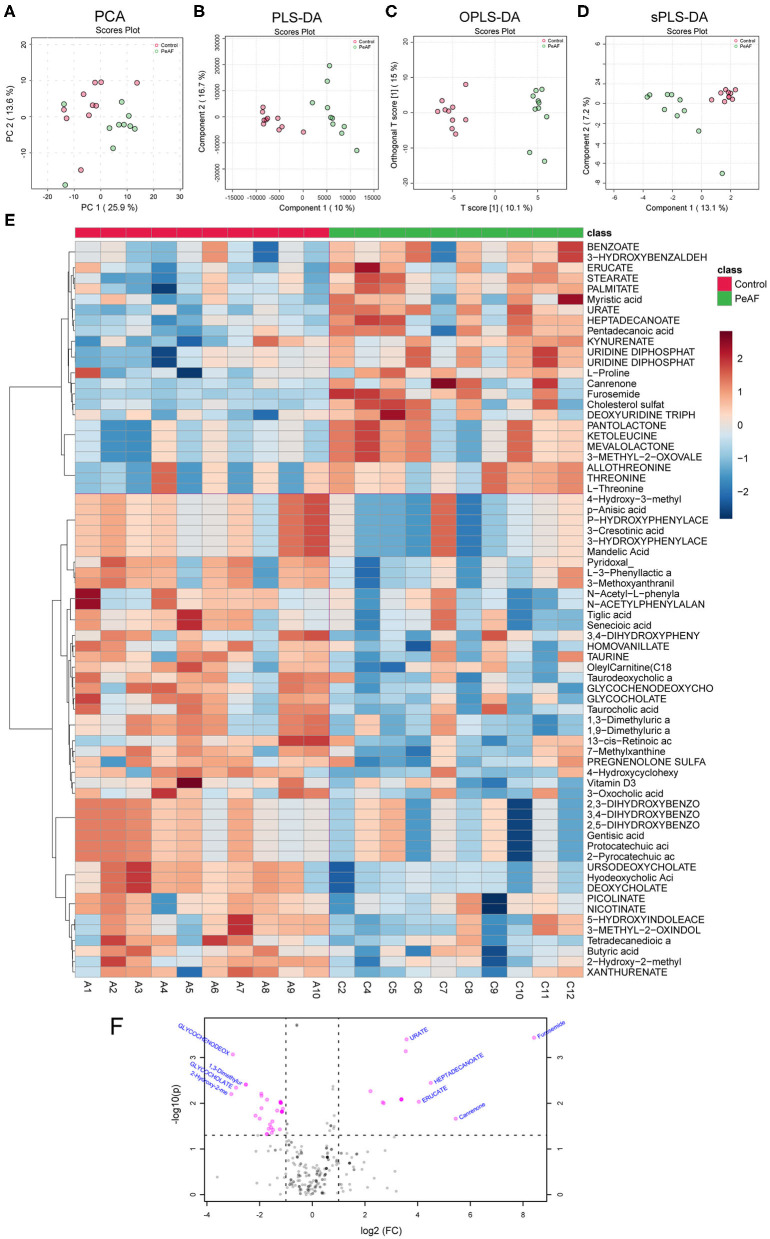
Differences in metabolites produced by the intestinal flora. **(A)** Principal component analysis (PCA). **(B)** Partial least squares discriminant analysis (PLS-DA). **(C)** Orthogonal partial least squares discriminant analysis (OPLS-DA). **(D)** Sparse partial least squares-discriminant analysis (SPLS-DA). **(E)** Cluster analysis of 70 different types of metabolites. **(F)** Volcanic plot of differential metabolites (|log2(FC)|>1, PeAF/control).

It is known that an essential way for the intestinal flora to exert positive and/or negative effects on the host is achieved through the metabolites produced by the flora. Therefore, we detected and analyzed the intestinal metabolites in the subjects in this study. As shown in Figure 6, the PCA score obtained by LC-MS metabonomics showed some degree of aggregation between the two groups (Figure 6A). Further analysis using PLS-DA (Figure 6B), OPLS-DA (Figure 6C), and SPLS-DA (Figure 6D) revealed that the control group samples were wholly separated from the PeAF group samples. We tested the abundances of 281 metabolites in total and analyzed the abundances of 70 different metabolites to generate an intestinal metabolite abundance heat map (Figure 6E). According to the multiple metabolite changes, we found that 48 metabolites were significantly increased, and 46 metabolites were significantly decreased in the PeAF group (Figure 6F).

### Functional Analysis of Differential Metabolite Abundances and Their Correlations With Myocardial Fibrosis

**Figure 7:**
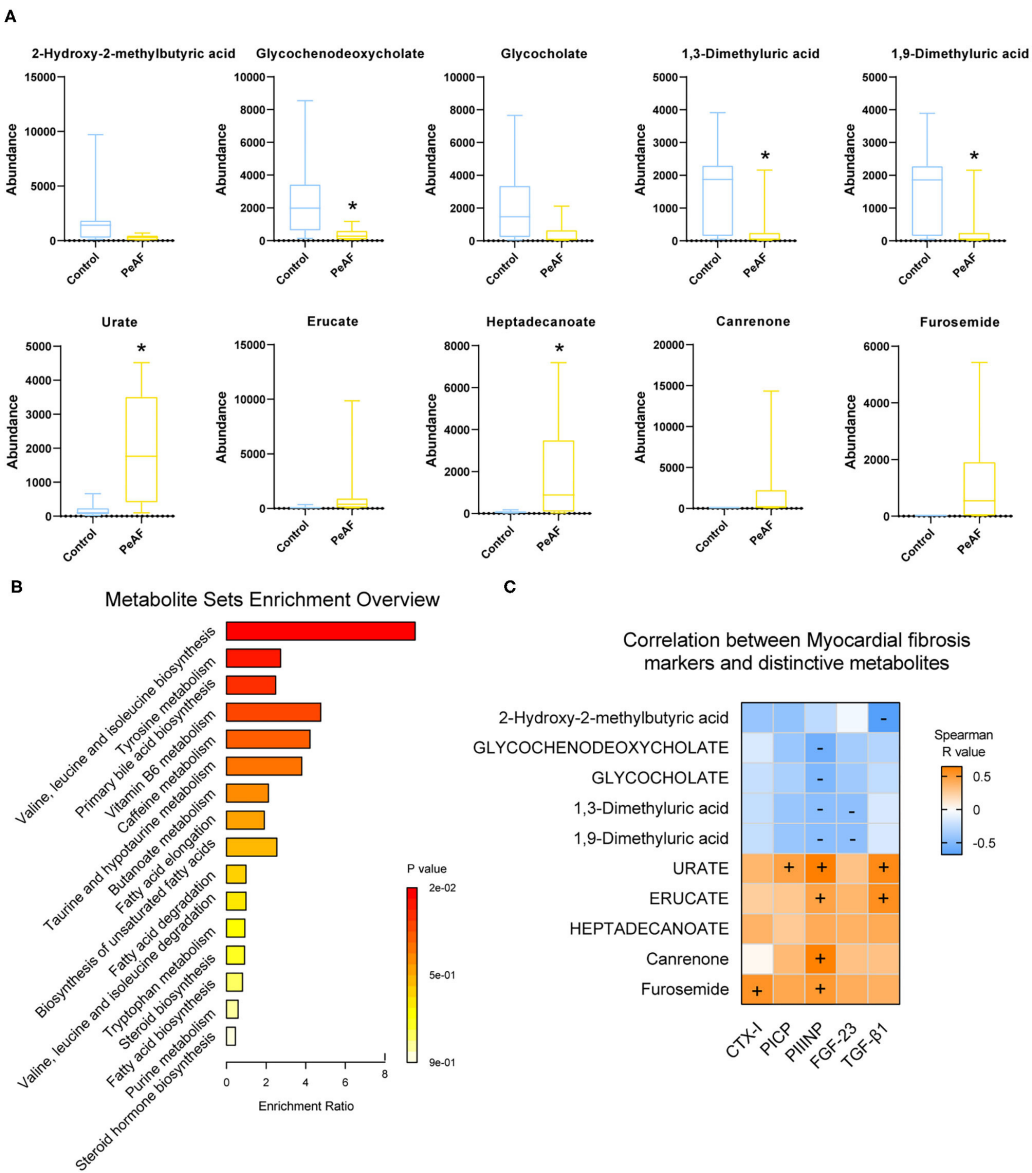
Functional analysis of differential metabolites and their correlation with myocardial fibrosis. **(A)** The top 5 differential metabolites that decreased and increased the most between control group and PeAF group. *Compared to the control group, *p* < 0.05. **(B)** Analysis of KEGG pathways of differential metabolites. **(C)** Correlation analysis of myocardial fibrosis markers and differential metabolites. “+” indicates positive correlation (orange), *p* < 0.05. “–" indicates negative correlation (blue), *p* < 0.05.

Based on the differences in the abundances of intestinal metabolites that we found, we performed *t*-tests to analyze the changes in metabolites in the control group and the PeAF group. The top 5 differential metabolites that decreased and increased the most in the two groups were shown in Figure 7A, which were 2-hydroxy-2-methylbutyric acid, Glycochenodeoxycholate, Glycocholate, 1,3-dimethyluric acid, 1,9-Dimmethyluric acid, Urate, Erucate, Heptadecanoate, Canrenone, and Furosemide. Among these, Glycochenodeoxycholate, 1,3-dimethyluric acid and 1,9-dimethyluric acid were significantly reduced in the PeAF group compared to the control group. The levels of Urate and Heptadecanoate were elevated considerably. Then, through KEGG pathway analysis, the top 16 signal pathways of the control group and the PeAF group were obtained (Figure 7B). The analysis performed using the KEGG pathway database (https://www.genome.jp/kegg/pathway.html), the functional metabolic pathways at the L1 level intestines PeAF patients changed significantly. In addition, 8 intestinal metabolites in the “valine, leucine, and isoleucine biosynthesis” pathway (L3 level) belonging to “amino acid metabolism” category (L2 level) were enriched in the PeAF patients (*p* = 1.07E-02); of these metabolites, C00671 [(S)-3-Methyl−2-oxopentanoic acid] and C00233 (4-Methyl-2-oxopentanoate) were significantly enriched in the intestines of the PeAF patients (Figure 7B). Meanwhile, a total of 42 plasma metabolites in the “tyrosine metabolism” pathway (L3 level) belonging to the “amino acid metabolism” category (L2 level) were enriched in the PeAF patients (*P* = 5.32E-02), of which metabolites C00628 (2,5-dihydroxybenzoate), C00642 (4-hydroxyphenylacetate), and C05582 (homovanillate) were significantly enriched in the intestines of patients in the PeAF group (Figure 7B). In addition, 46 intestinal metabolites in the “Primary bile acid biosynthesis” pathway (L3 level) belonging to the “Lipid metabolism” category (L2 level) were enriched (*P* = 6.66E-02), including C01921 (Glycocholate), C05466 (glycochenodeoxycholate), and C05122 (Taurocholate; Taurocholic acid), which were significantly enriched in the intestines of the PeAF patients (Figure 7B). These results indicate that the significantly enriched metabolites in the intestines of PeAF patients are related to the functional metabolic trends in amino acid metabolism and lipid metabolism pathways. Spearman's correlation analysis indicated that PIIINP significantly correlates with Urate, Erucate, Canrenone, and Furosemide (*p* < 0.05). In contrast, PIIINP has a significant negative correlation with 1,3-dimethyluric acid, 1,9-dimethyluric acid, Glycocholate, and glycochenodeoxycholate. FGF-23 is also significantly and negatively correlated with 1,3-dimethyluric acid and 1,9-dimethyluric acid. TGF-β1 was positively correlated with Urate and Erucate and negatively correlated with 2-hydroxy−2-methylbutyric acid. In addition, CTX-I, and PICP were positively correlated with Furosemide and Urate, respectively (Figure 7C). These results indicate that there may be a correlation between intestinal metabolism and myocardial fibrosis in PeAF patients.

## Discussion

In this study, the relationship between myocardial fibrosis and the composition of the intestinal flora and metabolic function in patients with PeAF was preliminarily explored. Atrial fibrillation (AF) is mediated by oxidative stress, neurohormonal activation, and inflammatory activation. Serum uric acid (SUA) is a surrogate indicator of oxidative stress (21). The reduction in the urinary albumin/creatinine ratio caused by high creatinine levels has also been associated with an increased risk of atrial fibrillation (22). In this study, similar results were obtained, and the levels of SUA and creatinine in the PeAF patients were significantly higher than those in the healthy population. Atrial fibrosis is a sign of the remodeling of the heart structure in patients with atrial fibrillation, and it is the basis for the development of atrial fibrillation. In addition, atrial fibrillation can aggravate atrial fibrosis. We found high levels of the cardiac fibrosis markers CIXT, PICP, PIIICP, FGF-23, and TGF-β1 in PeAF patients. Similar to CRE, FGF-23 levels have been previously reported to increase with decreased renal function in patients with atrial fibrillation (23). The health and normal functioning of the cardiovascular system and the renal system mainly depend on the crosstalk of the gut-kidney-cardiovascular system (24). It was also reflected in our research.

In recent years, the role of intestinal flora in cardiovascular disease has gradually attracted attention. Researchers have found that in many diseases, including atrial fibrillation, the structure and composition of the intestinal flora change to a certain extent. In this study, although the alpha diversity of the intestinal flora between healthy people and PeAF patients was no apparent difference, we observed significant differences in the relative indexes in terms of beta diversity, such as Bray-Curtis, weighted UniFrac and Anosim. It implies that the steady state of the intestinal microbial community structure of the PeAF population had been altered. Similarly, in a previous investigation, researchers found that the abundance of gut microbes in AF patients was lower than that of people without atrial fibrillation. However, they found no differences in gut microbial diversity between the two groups (25). This result may have been due to the limited number of samples. Firmicute has been identified as a beneficial bacterial phylum. In this study, at the phylum level, the abundance of Firmicutes was decreased sharply in the PeAF patients. In contrast, the abundance of Actinobacteria was significantly increased in the PeAF patients. Notably, the abundance of *Blautia* (Firmicutes phylum) has also been previously found to be significantly reduced in patients with chronic heart failure (26). *Rothia* (Actinobacteria phylum) has been found that was over-enriched in patients with certain diseases, such as pancreatic cancer and primary sclerosing cholangitis (27, 28). Previous studies have also indicated that a reduction in *Faecalibacterium prausnitzii* abundance is one of the fundamental characteristics of patients with chronic heart failure (29). However, at the species level, we observed an increase in the abundance of *g_Faecalibacterium_ASV_20* (*Faecalibacterium* genus) in the PeAF patients, which may be related to differences in the pathological development of different diseases. Kaburova et al. found that both PICP and PIIINP were significantly and negatively correlated with beneficial intestinal bacteria and significantly positively correlated with several potentially harmful bacteria in the intestine (30). We found that at the species level, *g_Faecalibacterium_ASV_20* (*Faecalibacterium* genus) and *g__Blautia_ASV_21* (*Blautia* genus) were significantly and positively correlated with PICP, PIIINP, and FGF-23 level, while *g__Blautia_ASV_5* (*Blautia* genus) was significantly and negatively correlated with PIIINP and FGF-23 level. This finding suggests that the specific intestinal flora of PeAF patients may be involved in the process of myocardial fibrosis.

The evidence obtained to date suggests that one of the potential mechanisms by which the intestinal flora has an impact on the host is by directly affecting the host through host-derived metabolites. For example, SCFAs produced through the metabolism of the intestinal flora can maintain the host's sugar, lipid and protein metabolism balance and reduce the occurrence and development of cardiovascular diseases (31). The metabolism of intestinal microorganisms determines the developmental direction of the host's health and illness to a certain extent. This study found that the intestinal metabolites Urate (i.e., Uric acid) and Heptadecanoate were increased significantly in the PeAF patients. Among them, uric acid is the end product of purine metabolism in humans. Studies have shown that Urate acts as a pro-oxidant at high concentrations, which induces AF to activate apoptosis and the immune system (32, 33). Kuo et al. found that in AF patients, the variation trend of some metabolites in feces and serum was consistent (13). Coincidentally, we also found a synchronous increase of uric acid in feces and serum in PeAF patients in this study. Thus, we hypothesize that gut microbial dysfunction at least partly affected the development of AF through internal circulation. In contrast, we found that the levels of 1,3/1,9-Dimethyluric acid and Glycochenodeoxycholate (i.e., Glycochenodeoxycholic acid) in the PeAF patients were significantly decreased. Glycochenodeoxycholate is a kind of conjugated primary bile acid. Although the abundance of Glycocholate (i.e., Glycocholic acid) did not differ significantly between the two groups, we observed a decreasing trend in the PEAF group. Interestingly, Glycochenodeoxycholate and Glycocholate belong to primary bile acids and secondary acids, respectively.

There is evidence that excessive lipid accumulation can lead to apoptosis and mitochondrial dysfunction and increase cardiac fibrosis (34, 35). In this study, we found through a KEGG pathway analysis that some enriched metabolites in PeAF patients are related to amino acid metabolism (valine, leucine, and isoleucine biosynthesis and tyrosine metabolism) and lipid metabolism (primary bile acid biosynthesis) pathways. Therefore, reducing amino acid metabolism and lipid metabolism damage may be an essential way to improve myocardial fibrosis patients with atrial fibrillation. In this study, Spearman's correlation analysis revealed that some metabolites were significantly related to myocardial fibrosis markers, especially PIIINP. Therefore, we speculate that the changes in the composition and structure of the specific flora of PeAF patients may trigger certain metabolic changes, and the resulting disruption in intestinal homeostasis may be a strong promoter that accelerates the process of myocardial fibrosis in PeAF patients.

We must admit that our study also has some limitations. On the one hand, the sample size included in this study was limited. It resulted in some changes that were not significantly different between the two groups (although some trends were observed). On the other hand, there was age bias between the two groups. Some of the differences in gut microbiota between individuals may be caused by age (36). Although we cannot completely rule out age bias in this study, we are collecting more clinical samples. We will validate our findings in larger cohorts and try to minimize any possible differences between groups due to age.

## Conclusion

In summary, our research shows that the occurrence of atrial fibrillation is accompanied by a certain degree of intestinal flora disorder. In addition, the degree of cardiac fibrosis in patients with atrial fibrillation is closely related to the abundance and metabolic function of particular intestinal flora. These microbes may directly or indirectly participate in cardiac fibrosis through metabolic pathways in patients with atrial fibrillation. The results provide new insights into the relationship between atrial fibrillation-myocardial fibrosis and intestinal flora.

## Data Availability Statement

The datasets presented in this study can be found in online repositories. The names of the repository/repositories and accession number(s) can be found at: https://www.ncbi.nlm.nih.gov/sra/PRJNA728204.

## Ethics Statement

The studies involving human participants were reviewed and approved by the Ethic Committee of Xiangya Hospital Central South University (202004176). The patients/participants provided their written informed consent to participate in this study.

## Author Contributions

FL and LL designed the research and performed the research. JS and RL analyzed the data. All authors contributed to the writing and revisions and reviewed the manuscript.

## Conflict of Interest

The authors declare that the research was conducted in the absence of any commercial or financial relationships that could be construed as a potential conflict of interest.

## Publisher's Note

All claims expressed in this article are solely those of the authors and do not necessarily represent those of their affiliated organizations, or those of the publisher, the editors and the reviewers. Any product that may be evaluated in this article, or claim that may be made by its manufacturer, is not guaranteed or endorsed by the publisher.
